# Structural basis for heme-dependent NCoR binding to the transcriptional repressor REV-ERBβ

**DOI:** 10.1126/sciadv.abc6479

**Published:** 2021-01-27

**Authors:** Sarah A. Mosure, Timothy S. Strutzenberg, Jinsai Shang, Paola Munoz-Tello, Laura A. Solt, Patrick R. Griffin, Douglas J. Kojetin

**Affiliations:** 1 Department of Integrative Structural and Computational Biology, The Scripps Research Institute, Jupiter, FL 33458, USA.; 2 Skaggs Graduate School of Chemical and Biological Sciences, The Scripps Research Institute, Jupiter, FL 33458, USA.; 3 Department of Immunology and Microbiology, The Scripps Research Institute, Jupiter, FL 33458, USA.; 4 Department of Molecular Medicine, The Scripps Research Institute, Jupiter, FL 33458, USA.

## Abstract

Heme is the endogenous ligand for the constitutively repressive REV-ERB nuclear receptors, REV-ERBα (NR1D1) and REV-ERBβ (NR1D2), but how heme regulates REV-ERB activity remains unclear. Cellular studies indicate that heme is required for the REV-ERBs to bind the corepressor NCoR and repress transcription. However, fluorescence-based biochemical assays suggest that heme displaces NCoR; here, we show that this is due to a heme-dependent artifact. Using ITC and NMR spectroscopy, we show that heme binding remodels the thermodynamic interaction profile of NCoR receptor interaction domain (RID) binding to REV-ERBβ ligand-binding domain (LBD). We solved two crystal structures of REV-ERBβ LBD cobound to heme and NCoR peptides, revealing the heme-dependent NCoR binding mode. ITC and chemical cross-linking mass spectrometry reveals a 2:1 LBD:RID stoichiometry, consistent with cellular studies showing that NCoR-dependent repression of REV-ERB transcription occurs on dimeric DNA response elements. Our findings should facilitate renewed progress toward understanding heme-dependent REV-ERB activity.

## Teaser

Structural and biochemical evidence resolves previous contradictory findings for heme-dependent NCoR recruitment to REV-ERBβ.

## INTRODUCTION

Nuclear receptors (NRs) are a superfamily of transcription factors that evolved to bind endogenous small-molecule ligands (*1*). Defining the molecular basis for NR regulation by their natural ligands provides important insight into how extracellular and intracellular signals are transmitted into changes in gene expression. This information helps identify processes that may be dysregulated in disease and informs the design of synthetic NR ligands with therapeutic potential.

The REV-ERBs, REV-ERBα (NR1D1) and REV-ERBβ (NR1D2), are closely related NRs with critical roles in mammalian physiology, including maintenance of the circadian rhythm, metabolic processes, and immune function (*2*, *3*). The REV-ERBs are unique among NRs because their ligand-binding domains (LBDs) lack the C-terminal activation function–2 (AF-2) helix 12 important for binding transcriptional coactivator proteins, suggesting that the REV-ERBs should interact exclusively with transcriptional corepressor proteins and solely repress transcription (*4*, *5*). This theory is supported by evidence that the REV-ERBs constitutively repress target genes (*6*).

The iron-centered porphyrin heme has been identified as the endogenous REV-ERB ligand (*7*, *8*). Cell-based evidence suggests that the REV-ERBs require heme to interact with the transcriptional corepressor protein NR corepressor-1 (NCoR) and repress transcription (*7*–*9*). Structural data would help establish that heme directly produces a REV-ERB LBD conformation that enhances NCoR binding. However, published biochemical and structural studies contradict cell-based evidence (*7*–*11*). In fluorescence-based biochemical assays, heme was shown to dose-dependently displace NCoR interaction domain (ID) peptides from the REV-ERBs (*7*–*11*). Furthermore, comparison of REV-ERB LBD crystal structures bound to either heme alone or an NCoR ID peptide alone indicated that the heme-bound conformation would directly clash with the NCoR-bound conformation (fig. S1) (*10*–*13*). Thus, the conflicting biochemical, structural, and cellular data have cast doubt on whether heme directly promotes REV-ERB interaction with NCoR and generally prevented progress toward understanding the molecular basis for REV-ERB activity.

Here, we show that heme-dependent fluorescence assay artifacts have led to an inaccurate conclusion that heme physically displaces NCoR from the REV-ERBs. Using two fluorescence-independent methods, isothermal titration calorimetry (ITC) and nuclear magnetic resonance (NMR) spectroscopy, we show that heme directly increases REV-ERBβ LBD affinity for one of two ID motif peptides within the NCoR receptor ID (RID). To support this finding, we solved two crystal structures of REV-ERBβ LBD cobound to heme and NCoR ID peptides, demonstrating the structural basis for heme-dependent NCoR binding. ITC and chemical cross-linking mass spectrometry (XL-MS) analysis of LBD interaction with a RID construct containing both ID motifs demonstrates a 2:1 LBD:RID stoichiometry where heme alters the interaction thermodynamics and facilitates cooperative RID binding. Collectively, our results provide biochemical and structural data that support an updated model for how heme regulates NCoR recruitment and transcriptional repression by REV-ERBβ in cells.

## RESULTS

### Heme and NCoR peptides cobind to the REV-ERBβ LBD

**Fig. 1:**
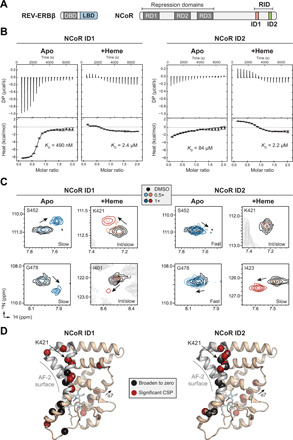
Heme directly promotes NCoR ID2 peptide binding to REV-ERBβ LBD. (**A**) REV-ERBβ and NCoR domain architecture. REV-ERBβ domains include DNA binding domain (DBD) and LBD. NCoR domains include repression domains (RDs) and the RID encompassing two ID motifs (ID1 and ID2). (**B**) Representative ITC thermograms and fitted curves of REV-ERBβ LBD titrated with NCoR ID1 or ID2 peptide in the presence or absence of heme with calculated binding affinities inset in each plot (error bars, uncertainty in each injection calculated by the NITPIC algorithm); each panel is representative of two or more independent experiments. (**C**) Representative peaks from 2D [^1^H,^15^N]-TROSY-HSQC spectra of ^15^N-labeled REV-ERBβ LBD with (orange/red peaks) or without heme (blue peaks) titrated with NCoR ID1 or ID2 peptide. (**D**) Residues with the largest chemical shift perturbation (CSP) changes or line broadening in the 2D [^1^H,^15^N]-TROSY-HSQC spectra with addition of 2× ID1 or ID2 peptide [relative to the dimethyl sulfoxide (DMSO) vehicle spectrum] were mapped onto the crystal structure of heme-bound REV-ERBβ LBD [Protein Data Bank (PDB) 3CQV]. ppm, parts per million. DP, differential power.

REV-ERB LBD interacts with NCoR via two ID motifs present within the RID (Fig. 1A) (*14*). We and others have observed that heme dose-dependently reduces REV-ERB LBD interaction with peptides derived from NCoR ID motifs in fluorescence-based assays (fig. S2A) (*7*–*11*). However, because heme quenches fluorophores across a broad range of emissions (fig. S2C) (*15*–*17*), we hypothesized that the decrease in signal in fluorescence-based peptide binding assays is due to heme quenching the fluorescent signal itself instead of heme physically displacing ID peptides from the LBD (fig. S2D). To test how heme affects REV-ERBβ LBD affinity for NCoR ID peptides using a fluorescence-independent method, we performed ITC to obtain binding affinity and thermodynamic parameters of binding (Fig. 1B, Table 1, and fig. S3). Ligand-free (apo) REV-ERBβ LBD exhibited high affinity for ID1 peptide and low affinity for ID2 peptide. Heme decreased LBD affinity for the ID1 peptide but increased LBD affinity for ID2 peptide, effectively equalizing the ID1 and ID2 peptide affinities. Heme also remodeled the thermodynamic profiles of peptide binding from enthalpically driven and exothermic to entropically driven and endothermic. These data provide the first biochemical evidence that heme does not inhibit NCoR binding and can increase LBD affinity for an NCoR peptide.

**Table 1:** Thermodynamic parameters of NCoR ID peptide binding to REV-ERBβ LBD. *K*_D_, binding affinity; Δ*G*, free energy of binding; Δ*H*, enthalpy of binding; temperature (*T*), constant at 25°C; and entropy (Δ*S*) of binding.

	**NCoR ID1 peptide**		**NCoR ID2 peptide**	
	**Apo LBD**	**+Heme** **LBD**	Apo LBD*	**+Heme** **LBD**
*K*_D_ (μM)	0.49 (95% ^†^CI: 0.40 to 0.61)	2.4 (95% CI: 1.0 to 5.7)	84 (95% CI: ^‡^n.d.)	2.2 (95% CI: 1.6 to 3.1)
Δ*G* (kcal/mol)	−8.6 (95% CI: −8.7 to −8.5)	−7.7 (95% CI: −8.2 to −7.2)	−5.6 (95% CI: n.d.)	−7.7 (95% CI: −7.9 to −7.5)
Δ*H* (kcal/mol)	−7.7 (95% CI: −8.0 to −7.5)	1.7 (95% CI: 1.4 to 2.0)	−9.7 (95% CI: n.d.)	3.0 (95% CI: 2.8 to 3.3)
*T*Δ*S* (kcal/mol)	0.88 (95% CI: 0.72 to 1.0)	9.3 (95% CI: 9.2 to 9.6)	−4.2 (95% CI: n.d.)	10.7 (95% CI: 10.7 to 10.8)

*Values are fitted estimates due to low *c* value.†Confidence interval (CI) calculated using SEDPHAT analysis software.

‡Not determined (n.d.) due to low *c* value.

To support our ITC results, we performed solution NMR structural footprinting by titrating NCoR ID1 or ID2 peptide into ^15^N-labeled REV-ERBβ LBD. For this analysis, we used backbone NMR chemical shift assignments that we previously reported for apo LBD (*18*) and collected three-dimensional (3D) NMR data to obtain backbone NMR chemical shift assignments for heme-bound LBD (fig. S4). Titration of the peptides revealed localized NMR chemical shift changes (i.e., peak movements) in 2D [^1^H,^15^N]-TROSY-HSQC (transverse relaxation optimized spectroscopy–heteronuclear single-quantum coherence) NMR spectra (fig. S5), confirming that the ID peptides bind to apo and heme-bound LBD. To qualitatively assess the strength of each interaction, we analyzed the exchange regime of NMR chemical shift changes on the NMR time scale. Consistent with their ITC-derived affinities, titration of ID1 and ID2 peptide into apo LBD (Fig. 1C) revealed slow and fast exchange events indicative of strong and weak binding, respectively. Titration of both peptides into heme-bound LBD revealed a mixture of peak movements in slow and intermediate exchange, consistent with the moderate affinities (~2 μM) of these interactions measured by ITC.

Next, we used differential NMR analysis to structurally map the ID1 and ID2 peptide binding surfaces in the heme-bound LBD. In other NRs, the AF-2 coregulator interaction surface is formed by the physical interaction of helix 12 with a surface formed by helices 3 to 5 within the LBD. REV-ERBβ lacks helix 12 but contains the surface formed by helices 3 to 5 including a conserved lysine residue (K421) on helix 3 important for corepressor interaction (*19*). We quantitatively analyzed chemical shift perturbations (CSPs) and line broadening caused by peptide binding in 2D [^1^H,^15^N]-TROSY-HSQC NMR spectra (Fig. 1D and fig. S6). AF-2 surface residues including K421 showed the largest CSP changes and line broadening, indicating that the peptides bind at the AF-2 surface. We also observed binding effects on helix 7, which may be attributed to a peptide-induced allosteric conformational change due to interactions between the heme propionate group and G480 on helix 7 (fig. S7).

### Crystal structures of REV-ERBβ LBD cobound to heme and NCoR peptides

**Fig. 2:**
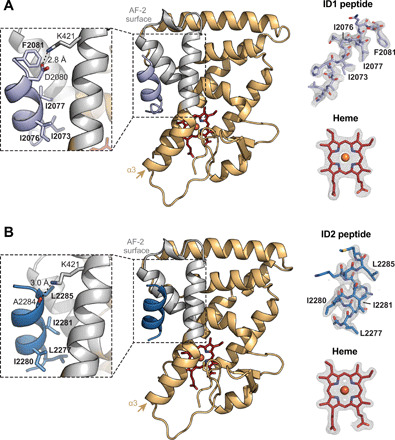
Crystal structures of REV-ERBβ LBD cobound to heme and NCoR ID peptides. (**A**) Structure of REV-ERBβ LBD (light orange cartoon with the AF-2 surface in gray) cobound to heme (red sticks) and NCoR ID1 peptide (light purple cartoon) (PDB 6WMQ). (**B**) Structure of REV-ERBβ LBD (light orange cartoon with the AF-2 surface in gray) cobound to heme (red sticks) and NCoR ID2 peptide (dark blue cartoon) (PDB 6WMS). Insets to the left of the structures highlight the ID peptide CoRNR box motif residues (in bold) and conserved charge clamp interaction with K421. Omit maps (2*F*_o_ − *F*_c_, contoured at 1σ) for the ID peptides and heme are shown to the right of the structures with CoRNR box motif residues indicated.

Collectively, our ITC and NMR data showed that heme-bound REV-ERBβ LBD can bind NCoR ID motif peptides at the AF-2 surface. To determine the structural basis of this interaction, we solved crystal structures of REV-ERBβ LBD cobound to heme and NCoR ID1 peptide (2.6-Å resolution) or ID2 peptide (2.0-Å resolution) (Fig. 2, A and B, respectively, and table S1). In both structures, REV-ERBβ LBD crystallized with two molecules in the asymmetric unit with an NCoR ID peptide and heme molecule cobound to each LBD. The overall root mean square deviation (RMSD) of the complex in the asymmetric unit including LBD, heme, and NCoR ID peptide was 1.1 and 1.3 Å for the ID1- and ID2-bound structures, respectively, suggesting minimal conformational heterogeneity. The ID peptides bound to the AF-2 surface formed electrostatic interactions with the conserved charge clamp residue K421 on helix 3, confirming that the heme-dependent REV-ERBβ corepressor interaction is similar to other NRs (*19*).

We sought to understand the structural changes underlying heme and NCoR ID peptide cobinding to REV-ERBβ LBD. The overall heme-bound LBD conformations were largely unchanged by peptide binding, with average LBD RMSDs of 1.0 and 1.3 Å for the ID1- and ID2-bound structures, respectively, relative to the published heme-bound LBD structure (*11*). In the crystal structure of apo REV-ERBα LBD bound to NCoR ID1 peptide, an antiparallel β sheet is formed between the N terminus of the ID1 peptide and a β strand extension off helix 11 (*10*). In our heme-bound structure, this antiparallel β sheet is necessarily altered to accommodate cobinding with heme to prevent a clash between helix 3 and the peptide (fig. S8A). Although the antiparallel β sheet was lost, the AF-2 interaction with the α-helical region of the ID1 and ID2 peptides containing the I/LxxI/LI/LxxxI/L/F corepressor NR (CoRNR) box motif critical for corepressor binding was preserved.

The structural features in our heme and NCoR peptide cobound crystal structures provide insight into the peptide affinities obtained by ITC (Table 1). Heme binding inhibits formation of the antiparallel β sheet, which likely contributes to higher-affinity ID1 binding to the apo LBD, resulting in lower-affinity ID1 binding to heme-bound LBD. This conclusion is supported by a previous study showing that truncation of β sheet–forming ID1 residues reduced binding affinity for apo REV-ERBα with a decrease nearly identical to the heme-dependent reduction in ID1 affinity that we determined by ITC (*13*). Furthermore, our crystal structures show similar binding modes for ID1 and ID2 (fig. S8B), providing a structural rationale for how these peptides display similar affinities (~2 μM) for heme-bound REV-ERBβ LBD.

### Heme promotes cooperative binding of NCoR RID

**Fig. 3:**
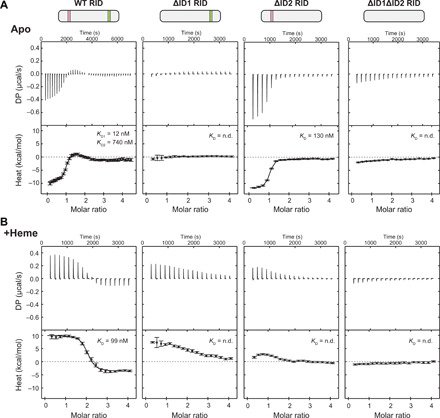
Heme remodels REV-ERBβ LBD interaction with an NCoR RID construct composed of both ID motifs. Representative ITC thermograms and fitted curves of apo (**A**) or heme-bound (**B**) REV-ERBβ LBD and NCoR RID constructs; data are representative of at least two experiments per condition (error bars, uncertainty in each injection calculated by the NITPIC algorithm). Calculated binding affinities are shown for the fitted datasets.

Our ITC data showed that heme normalizes NCoR ID1 and ID2 peptide affinity for REV-ERBβ LBD such that both peptides bind heme-bound LBD with approximately equal affinity. We hypothesized that this might be advantageous for a cooperative interaction with the entire NCoR RID containing both ID motifs (*20*). ITC analysis of wild type (WT) RID interaction with apo LBD revealed a binding curve with two transitions yielding affinities of 12 and 740 nM when fit to a two-site interaction model (Fig. 3A and Table 2). This indicates that two apo LBDs interact with one RID, most likely with positive cooperativity, given that the two fitted RID affinities are higher than the affinities for individual ID peptides (Table 1). On the basis of our findings with ID peptides, we reasoned that ID1 and ID2 would mediate the higher- and lower-affinity binding events, respectively. To test this, we mutated the ID1 (ΔID1), ID2 (ΔID2), or both (ΔID1ΔID2) motifs within the RID and tested them for binding by ITC. Consistent with the ID1 and ID2 peptide affinities and a role for positive cooperativity, the ΔID1 and ΔID1ΔID2 mutants abolished binding, while ΔID2 eliminated the second, weaker transition and modestly reduced the affinity of the first site.

**Table 2:** Thermodynamic parameters of NCoR RID binding to REV-ERBβ LBD.

	**Apo LBD**		**^+^Heme LBD**
	**WT RID**	**ΔID2 RID**	**WT RID**
*K*_D_ (nM)	Site 1: 12 (95% *CI: 1.4 to 56)	130 (95% CI: 100 to 180)	99 (95% CI: 46 to 460)
	Site 2: 740 (95% CI: 380 to 1500)		
Δ*G* (kcal/mol)	Site 1: −10.8 (95% CI: −12.1 to −9.9)	−11.1 (95% CI −11.5 to −10.8)	−9.6 (95% CI: −10.0 to −8.6)
	Site 2: −8.4 (95% CI: −8.8 to −8.0)		
Δ*H* (kcal/mol)	Site 1: −8.3 (95% CI: −9.1 to −7.7)	−9.4 (95% CI −9.6 to −9.2)	1.7 (95% CI: 1.4 to 2.0)
	Site 2: 3.2 (95% CI: 2.4 to 4.6)		
^†^*T*Δ*S* (kcal/mol)	Site 1: 2.5 (95% CI: 2.2 to 3.0)	−1.8 (95% CI −1.9 to −1.6)	11.2 (95% CI: 10.7 to 11.4)
	Site 2: 12 (95% CI: 11 to13)		

*CI calculated using SEDPHAT analysis software.

†Temperature (*T*), which was constant at 25°C, and entropy (Δ*S*) of binding.

We next determined the effect of heme on RID binding to LBD (Fig. 3B and Table 2). ITC analysis of RID interaction with heme-bound LBD produced a binding profile with a single transition around two equivalents of titrated LBD. This indicates that there are two binding events with similar binding enthalpies, which qualitatively agrees with our ID peptide ITC data showing that heme equalizes peptide affinities and enthalpies of binding. Furthermore, RID binding to heme-bound LBD must involve positive cooperativity since the fitted affinity (99 nM) is much stronger than the individual ID1 and ID2 peptide motifs (~2 μM) (Table 1). We also used our RID mutants to confirm the apparent 2:1 heme-bound LBD:RID binding profile. Whereas the ΔID1ΔID2 mutant eliminated binding, the ΔID1 and ΔID2 single mutants altered the binding profile but still showed two, albeit qualitatively weaker, binding events. The two-site transition profile of the single mutant data suggests that binding at the unmutated ID motif may partially rescue binding of mutated ID motif, which would occur only if cooperative binding is involved.

Given the evidence for positive cooperativity in RID binding to heme-bound LBD, we attempted to fit the WT RID data to a two-site model incorporating cooperativity. However, this objective was not straightforward, given that the heme-bound LBD binding to WT RID lacks distinct ITC binding transitions (i.e., identical enthalpic contributions to binding) such that cooperative and noncooperative fitting is indistinguishable on the basis of fit. Because of these complications, it was possible to produce several identical-looking fits with vastly different *K*_D_ [dissociation constant (binding affinity)] values and cooperativity factors. Thus, while we cannot conclude whether heme directly enhances RID binding, it is apparent that heme profoundly remodels the RID binding mechanism, most likely by facilitating positive cooperativity of ID motif binding. Together, these data show that RID binding to heme-bound LBD involves positive cooperativity, remodels the thermodynamics of the RID binding mechanism, and equalizes the ID motif affinities such that the RID may bind with higher overall affinity relative to apo LBD.

### Heme stabilizes a trimeric REV-ERBβ:NCoR complex

Our NCoR RID ITC data indicated that REV-ERBβ LBD and NCoR RID form a 2:1 complex. To further support this stoichiometry, we performed chemical cross-linking analysis of apo or heme-bound LBD ± RID using DSSO (disuccinimidyl sulfoxide), a cross-linker that cross-links lysine residues as well as serine, threonine, and tyrosine residues (fig. S9, A and B). By SDS–polyacrylamide gel electrophoresis (SDS-PAGE), we observed a weak dimer band in LBD-only samples that was unchanged by heme, which is consistent with ITC control data (fig. S3B), indicating that the LBD is predominantly monomeric and heme is insufficient to induce dimerization in the absence of RID. Addition of RID to LBD ± heme produced two bands with molecular weights consistent with 1:1 and 2:1 LBD:RID complexes. These data confirm the ITC stoichiometries, showing that two equivalents of apo and heme-bound LBD can interact with the RID.

**Fig. 4:**
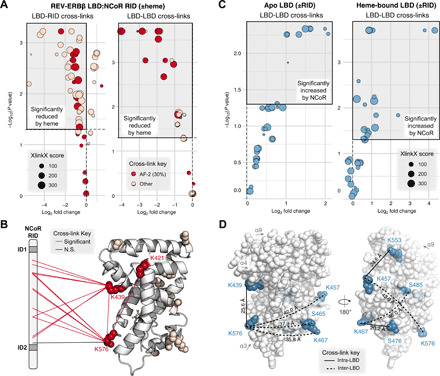
XL-MS analysis of heme-dependent LBD:RID interactions. (**A**) Differential analysis of LBD-LBD and LBD-RID cross-links in REV-ERBβ LBD + NCoR RID samples prepared in the presence or absence of heme. AF-2 cross-links involve residues K421, K439, or K576. The top left quadrant (shaded gray box) highlights cross-links significantly reduced by heme (*P*_adj_ < 0.05). (**B**) AF-2 (red) or other (pink) residues involved in cross-links significantly reduced by heme [gray box in (A)] are shown as spheres mapped to PDB 3CQV. LBD-LBD and LBD-RID cross-links involving AF-2 residues are indicated with red or black lines (all but one was significantly reduced by heme). N.S., not significant. (**C**) Differential analysis of LBD-LBD cross-links in apo (left) or heme-bound (right) REV-ERBβ LBD in the presence or absence of NCoR RID. The top right quadrant (shaded gray box) indicates cross-links significantly increased by RID binding. (**D**) LBD-LBD cross-links increased by RID in both apo and heme-bound LBD (blue) mapped to PDB 3CQV. Cross-links likely to be inter-LBD (SASD >25 Å) are indicated with dashed lines, while cross-links likely to be intra-LBD (SASD ≤25 Å) are indicated with solid lines; calculated SASDs are shown next to each line.

We next performed XL-MS to gain quantitative insight into the effect of heme binding on the LBD and RID interaction (fig. S9C). When comparing cross-links in LBD:RID complexes ± heme, we found that heme binding reduced LBD-LBD and LBD-RID cross-links, particularly for residues in the AF-2 surface (Fig. 4, A and B, and fig. S9D). Since the SDS-PAGE bands for these were qualitatively similar and the quantitative LBD-RID XL-MS cross-link abundances were reduced but not eliminated, it is unlikely that the reduction in cross-links is due to heme weakening the LBD-RID interaction. Instead, another interpretation of these findings that is consistent with our ITC data is that the higher-affinity (slower *K*_off_) interaction in the presence of heme reduces access of the DSSO cross-linker to LBD residues that are in direct contact with the RID.

There are two mechanisms by which RID binding could produce a 2:1 LBD:RID stoichiometry: one in which the LBD forms a dimer upon binding RID, which could explain the cooperativity observed by ITC, and another where the LBDs bind separately at each ID motif and do not interact. To assess these mechanisms using XL-MS, we determined how LBD-LBD cross-links in apo LBD and heme-bound LBD are affected upon binding RID (Fig 4C and fig. S9, E and F). We found that RID binding enhanced LBD-LBD cross-links. Solvent accessible surface distance (SASD) measurements revealed that many of the residues involved in the enhanced LBD-LBD cross-links are not likely to occur from intra-LBD (within monomer) cross-links because the distance between these residues is greater than the upper cutoff (~25 Å) for DSSO cross-linked residues (Fig. 4D). Instead, the SASD measurements indicated that these enhanced LBD-LBD cross-links are likely mediated by RID binding–induced inter-LBD interactions (dimerization). Because LBD alone did not form a robust dimer band by SDS-PAGE (fig S9, A and B), the increased cross-links in this context suggests that RID binding brings two LBDs in proximity, facilitating LBD-LBD contacts between two LBD molecules. Some of these inter-LBD cross-links involve residues within or on helix 7 and 11, which constitutes the canonical NR dimerization surface. However, other inter-LBD cross-links are within the β sheet region (fig. S9, E and F), which is part of a surface shown to mediate noncanonical dimerization of androgen receptor (*21*). Since XL-MS is sensitive to transient protein-protein interactions (*22*), it is possible that LBD dimers formed upon RID binding dynamically sample both canonical and noncanonical dimerization conformations.

## DISCUSSION

The closely related REV-ERB NRs, REV-ERBα and REVERBβ, are transcriptional repressors whose natural ligand is the iron-centered porphyrin heme. The role of heme in regulating REV-ERB activity has been unclear because of contradictory cell-based and structural evidence, suggesting that the heme-bound LBD is not capable of binding NCoR (*7*–*11*). Here, we resolve this conflict by showing that heme binding does not displace NCoR RID but instead remodels the interaction thermodynamics and facilitates positive cooperativity of binding. Collectively, our findings should facilitate renewed efforts toward understanding the molecular basis of heme-regulated REV-ERB function in health and disease.

**Fig. 5:**
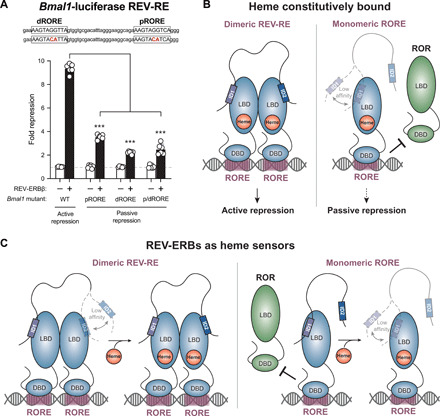
Stoichiometric principles of REV-ERB–dependent transcriptional repression. (**A**) *Bmal1-*luciferase transcriptional repression assay. Repression of luciferase activity by REV-ERBβ relative to empty vector was assessed for different *Bmal1* REV-RE promoter constructs including WT and mutant variants where the proximal (pRORE), distal (dRORE), or both (p/dRORE) response elements in the *Bmal1* REV-RE were mutated. Bars show means ± SD with individual technical replicates shown. Data are representative of *n* = 3 individual experiments; ***P < 0.001 by Student’s *t* test. (**B** and **C**) Models for the effect of heme on REV-ERB activity at dimeric REV-RE or monomeric RORE sites, assuming that (B) heme is constitutively bound to REV-ERBs in cells or (C) heme functions as a signaling molecule and REV-ERBs are not always bound to heme on DNA.

Our ITC and XL-MS experiments revealed that REV-ERBβ LBD and NCoR RID form a 2:1 complex. This observation is compelling in light of previous studies showing that the REV-ERBs only recruit NCoR and engage in active (corepressor-dependent) transcriptional repression on dimeric DNA response elements (REV-RE or REV-DR2) composed of two individual ROR (retinoic acid-related orphan receptor) response elements (ROREs) (*6*, *20*, *23*). Using a transcriptional reporter assay, a previous study showed that an intact dimeric REV-RE is required for REV-ERBα–mediated active repression of the *Bmal1* promoter (*6*). We confirmed that the same principles apply to REV-ERBβ (Fig. 5A). REV-ERBβ robustly repressed the WT *Bmal1* construct in a manner consistent with NCoR-dependent active repression (approximately ninefold repression), while repression of the mutant *Bmal1* constructs where one or both DNA binding sites within the REV-RE sequence was mutated was significantly reduced by more than half (approximately threefold repression) (*6*). The weak repression that occurs at monomeric RORE sites is thought to occur through a passive mechanism in which REV-ERBs do not recruit NCoR but block target gene activation by competing with the transcriptionally activating ROR NRs for binding at the same monomeric ROREs (*24*, *25*).

These data indicate that a dimeric REV-RE site is required for REV-ERBs to recruit NCoR on DNA, but cellular studies have suggested that heme binding is also necessary for REV-ERBs to recruit NCoR (*7*–*9*). These observations together with our structural findings here suggest two potential models for heme regulation of REV-ERB transcriptional repression. A model that is best supported by both our data and published data is one in which REV-ERBs are constitutively bound to heme in cells (Fig. 5B). In this scenario, NCoR is recruited to dimeric REV-RE sites and interacts with two heme-bound REV-ERBs with high affinity and positive cooperativity. At monomeric ROREs, where only a single heme-bound REV-ERB is available, the lack of cooperative binding may weaken NCoR affinity such that NCoR recruitment may not occur. Thus, our data fully explain cell-based observations, given a model in which REV-ERBs are always bound to heme on DNA.

Although several studies have suggested that REV-ERBs may be constitutively bound to heme in cells (*6*, *9*), others have suggested that REV-ERBs function as heme sensors (*26*) (Fig. 5C). In this scenario, structural and biochemical data indicate that the REV-ERBs may modestly interact with NCoR in the absence of heme via relatively high-affinity binding of the ID1 motif and weak binding of the ID2 motif. For REV-ERBs bound to dimeric REV-RE sites, heme binding could strengthen NCoR interaction by promoting cooperative binding of both ID motifs; at the same time, heme binding would be expected to displace NCoR from REV-ERBs bound to monomeric ROREs.

One caveat to our models is that our structural data here focuses on REV-ERBβ, so the relevance of our conclusions to REV-ERBα is not guaranteed. REV-ERBα and REV-ERBβ are thought to be functionally redundant, and cell-based studies have reported similarities including heme-dependent NCoR binding and overlapping target genes (*7*–*9*, *27*). Here, we further validated that the DNA-dependent repression mechanisms of REV-ERBα (*6*) and REV-ERBβ (Fig. 5A) are similar. Although current evidence strongly suggests that REV-ERBα and REV-ERBβ function similarly, future studies are nonetheless needed to confirm whether the heme-dependent structural mechanisms that we report here for REV-ERBβ also apply to REV-ERBα.

Together, our two models (REV-ERBs constitutively bound to heme versus REV-ERBs as heme sensors) synthesize existing cellular and structural evidence for heme- and DNA-dependent NCoR recruitment. However, we recognize that additional factors could contribute to heme-dependent NCoR recruitment, including signaling of diatomic gases and binding to other proteins such as ubiquitin ligases (*9*, *28*). Thus, future studies are warranted to address the roles of these additional factors, validate current models in the context of full-length REV-ERBs, and explore the interplay between DNA binding and heme-dependent NCoR interaction.

## MATERIALS AND METHODS

### Plasmids, reagents, and cell lines

The human REV-ERBβ LBD (residues 381 to 579) was previously cloned into the pET46 vector (*18*). The mouse NCoR RID (residues 1942 to 2208 in the X50 splice variant), which has 87% sequence identity to human NCoR RID (100% sequence identity in the ID motifs), was previously cloned into the pET32 vector (*29*). NCoR RID mutants were generated by site-directed mutagenesis: The ID1 CoRNR box motif was mutated from ICQIITQDF to TCQTTTQDF (ΔID1 and ΔID1ΔID2), and the ID2 CoRNR box motif was mutated from LEDIIRKAL to TEDTTRKAL (ΔID2 and ΔID1ΔID2), as these mutations were previously reported to disrupt CoRNR box binding (*10*, *30*). Heme (Sigma-Aldrich, no. 51280) was prepared either as a 1 mM stock in dimethyl sulfoxide (DMSO) stored at 4°C or 50 mM stock in 0.2 M NaOH (solutions prepared in 0.2 M NaOH were always made fresh immediately before the experiment), where indicated. NCoR ID1 peptide (RTHRLITLADHICQIITQDFARN) and NCoR ID2 peptide (DPASNLGLEDIIRKALMGSFDDK) were purchased with >95% from LifeTein with N-terminal amidation and C-terminal acetylation for stability and prepared as 50 mM stocks in DMSO stored at −80°C. Human embryonic kidney (HEK) 293T cells (American Type Culture Collection, no. CRL-11268) were cultured in Dulbecco’s modified eagle medium (DMEM) supplemented with 10% fetal bovine serum, 2 mM l-glutamine, and 1% penicillin-streptomycin at 37°C and 5% CO_2_ under standard culture conditions. The pcDNA3.1^+^ expression vector containing full-length human REV-ERBβ (*31*) and the pGL3-*Bmal1*-luciferase construct containing the *Bmal1* promoter (−816 to +99 of the mouse *Bmal1* promoter region) were used in HEK293T cell transfections. Site-directed mutagenesis was performed to mutate the proximal RORE (pRORE mutant), distal RORE (dRORE mutant), or both (p/dRORE mutant) in the pGL3-*Bmal1*-luciferase construct based on a previous study (*6*) where the ROREs were mutated to disrupt REV-ERBα activity.

### REV-ERBβ LBD expression and purification

Human REV-ERBβ LBD was expressed in BL21(DE3) *Escherichia coli* cells with an N-terminal hexahistidine (6xHis) tag separated by a 3C protease cleavage site. Expression was performed using auto-induction media: Cells were grown at 37°C for 5 hours, 30°C for 1 hour, and 18°C for 16 hours before harvesting by centrifugation. Pellets were resuspended in potassium phosphate lysis buffer [40 mM potassium phosphate (pH 7.4), 500 mM KCl, 15 mM imidazole, and 1 mM dithiothreitol (DTT)] supplemented with leupeptin, pepstatin A, lysozyme, and deoxyribonuclease (DNase) I and sonicated on ice. Lysed cells were centrifuged at 20,000*g* for 30 min at 4°C, and soluble lysate was filtered before immobilized metal affinity chromatography (IMAC) purification using 2 × 5 ml HisTrap columns (GE Healthcare) affixed to an ÄKTA pure. A 5-ml aliquot of 6xHis-tagged protein was purified by size exclusion chromatography using a Superdex 75 column equilibrated in time-resolved fluorescence energy transfer (TR-FRET) assay buffer [20 mM potassium phosphate (pH 7.4), 50 mM KCl, and 0.5 mM EDTA]; purified aliquots were stored at −80°C for TR-FRET assays. To cleave the 6xHis tag from the remaining protein, it was incubated overnight with 3C protease (generated in-house) via dialysis; then, protein was reloaded onto the HisTrap columns, and the flow-through was collected. Last, aggregates were removed by size exclusion chromatography using a Superdex 75 column and Hepes gel filtration buffer [20 mM Hepes (pH 7.4), 50 mM KCl, and 0.5 mM EDTA]. Greater than 95% purity was confirmed by SDS-PAGE. Protein was aliquoted and stored at −80°C.

### Crystallization, data collection, and structure determination

REV-ERBβ LBD was incubated with 2 molar equivalents of heme (prepared fresh at 50 mM in 0.2 M NaOH) overnight at 4°C before addition of 5 molar equivalents of either NCoR ID1 peptide or NCoR ID2 peptide. After overnight incubation at 4°C with peptide, REV-ERBβ LBD cobound to heme and peptide was buffer-exchanged into Hepes gel filtration buffer to remove unbound and concentrated to 15 mg/ml (ID2-bound) or 5 mg/ml (ID1-bound). Buffer-exchanged proteins were screened for conditions that produced crystals using the NR LBD (Molecular Dimensions), Structure (Molecular Dimensions), Index (Hampton Research), and PEG/Ion (Hampton Research) kits and sitting drop method (1-μl reservoir solution added to 1-μl drop of protein solution) at 22°C. REV-ERBβ LBD crystals cobound to heme and NCoR ID2 grew in 0.2 M Mg formate dihydrate and 20% (w/v) PEG 3350 (PEG/Ion), and REV-ERBβ LBD crystals cobound to heme and NCoR ID1 grew in 2.0 M ammonium sulfate, 0.1 M Na Hepes (pH 7.5), and 2% PEG 400 (Structure). Crystals in their respective condition were supplemented with 10% glycerol and flash-cooled in liquid nitrogen. X-ray diffraction data were collected at the Advanced Light Source synchrotron (Lawrence Berkeley National Laboratory) in 180 images with a 1° rotation per image. Data were processed, indexed, and scaled using Mosflm and Scala in CCP4 (Collaborative Computational Project No. 4) (*32*, *33*). Molecular replacement was performed using the program Phaser (*34*) in the Phenix software package (*35*) using the heme-bound REV-ERBβ LBD crystal structure as a search model [Protein Data Bank (PDB) 3CQV] (*11*). The structures were solved at 2.55 Å (for the ID1-bound structure) and 2.0 Å (for the ID2-bound structure). The NCoR ID1 and NCoR ID2 peptides were manually built into the unmodeled density using Coot (*36*). Subsequent iterations of automated refinement were performed using Phenix with *XYZ* coordinates, real-space individual B-factors, and occupancy parameters together with several cycles of manual modifications in Coot.

### Structural alignments and RMSD calculations

Structures were aligned, and RMSD values were calculated in PyMOL using the “align” command with cycles set to 0 (no outliers were removed). Waters were excluded from all alignments. For the alignment of the chains within the asymmetric units, LBD, heme, and peptide were aligned together as a single object. For alignment of the heme and peptide cobound structures with the published heme-bound structure, heme and LBD were included, while peptide was excluded. Both chains in the heme and NCoR ID peptide cobound REV-ERBβ LBD structures were aligned individually to the heme-bound REV-ERBβ LBD (which crystallized as monomer), and the average of the two values was reported. For alignment of the heme-bound REV-ERBβ LBD cobound to NCoR ID1 and ID2, the LBD, heme, and ID peptide were aligned together as a single object; the respective chains A and B were aligned, and the average RMSD of the two values was reported.

### Isothermal titration calorimetry

ITC experiments were performed using a MicroCal iTC200. All experiments were solvent-matched and contained 0.1% DMSO (peptide vehicle) and 0.2% 0.2 M solution of NaOH (heme vehicle) final concentrations. NITPIC software (*37*) was used to calculate baselines and integrate curves and prepare experimental data for fitting in SEDPHAT, which was used to generate final binding affinity and thermodynamic parameter measurements (*38*).Final figures were exported to GUSSI for publication-quality figure preparation (*39*).

NCoR ID1 and NCoR ID2 peptides were prepared at 50 μM in assay buffer [20 mM Hepes (pH 7.4), 50 mM KCl, 0.5 mM EDTA, and 5 mM tris(2-carboxyethyl)phosphine (TCEP)], and REV-ERBβ LBD was prepared at 500 μM. LBD in the syringe was titrated into ID peptide in the sample cell at 25°C with a 60-s delay between injections (2-μl intervals) with a mixing speed of 1200 rpm for a total of 20 injections (or 2 molar equivalents of LBD), unless otherwise noted below. For experiments with heme, 1.05 molar equivalents of heme prepared in 0.2 M NaOH at 50 mM were added to the REV-ERBβ LBD. Data were fit to A + B ↔ AB hetero-association model in SEDPHAT.

NCoR RID constructs (WT, ΔID1, ΔID2, or ΔID1ΔID2) were prepared in assay buffer at 15 μM, and REV-ERBβ LBD was prepared at 300 μM. REV-ERBβ LBD in the syringe was titrated into the RID in the sample cell as described above, with the exception that a total of 4 molar equivalents were titrated over 20 injections. For apo LBD into WT RID, 2 × 20 injections of 150 μM LBD were titrated into 15 μM RID, and data were concatenated using Concat ITC software (Malvern Panalytical) to better define the ITC curve, providing a more confident fitting of the second, weaker affinity transition. For apo LBD into WT RID, data were fit to A + B ↔ AB + B ↔ BA + A ↔ BAB, with two nonsymmetric sites, microscopic K model in SEDPHAT. For apo LBD into ΔID2, data were fit to an A + B ↔ AB hetero-association model in SEDPHAT. For heme-bound LBD into WT RID, data were fit to an A + B ↔ AB hetero-association model in SEDPHAT.

### Generation of isotopically labeled REV-ERBβ LBD

For generation of ^15^N-labeled REV-ERBβ LBD, protein was expressed in BL21(DE3) *E. coli* cells using M9 minimal media supplemented with ^15^NH_4_Cl (Cambridge Isotope Laboratories) induced at an optical density at 600 nm (OD_600_) of 0.6 with 0.5 mM isopropyl-β-d-thiogalactopyranoside (IPTG) for 16 hours at 18°C. For generation of ^2^H-,^15^N-,^13^C-labeled REV-ERBβ LBD (~70% deuteration) for peak assignment experiments, cultures were first grown at 37°C in 1 liter of LB media until an OD_600_ of 0.6 before the cells were pelleted and resuspended in 0.5 liters of M9 minimal media prepared in D_2_O supplemented with ^13^C-d-glucose and ^15^NH_4_Cl (Cambridge Isotope Laboratories). After 1-hour recovery at 37°C, expression was induced with 0.5 mM IPTG for 16 hour at 37°C before harvesting by centrifugation. The purifications were performed as described for unlabeled protein.

### NMR spectroscopy

To generate heme-bound protein, 1.25 molar equivalents of heme prepared in 0.2 M NaOH as a 50 mM stock were added to ^15^N-labeled REV-ERBβ LBD (200 μM) or ^2^H-,^15^N-,^13^C-labeled REV-ERBβ LBD (1 mM) in Hepes buffer [20 mM Hepes (pH 7.4), 50 mM NaCl, and 0.5 mM EDTA]. NMR data were collected at 298 K on a Bruker 700-MHz NMR system equipped with a QCI CryoProbe, processed using NMRFx (*40*), and analyzed using NMRViewJ (*41*). TROSY-based 3D HNCO, HNCA, HN(CA)CB, HN(COCA)CB, HN(CO)CA, and HN(CA)CO experiments were collected using the heme-bound ^2^H-,^15^N-,^13^C-labeled-REV-ERBβ LBD for backbone NMR chemical shift assignment. 2D [^1^H,^15^N]-TROSY-HSQC data were collected for heme-bound ^15^N-labeled REV-ERBβ LBD at 298 K with addition of NCoR ID1 or ID2 peptides prepared as 50 mM stock in DMSO and analyzed in NMRViewJ. CSP analysis was performed using 2D [^1^H,^15^N]-TROSY-HSQC heme-bound REV-ERBβ spectrum. Transfer of peak assignments from the vehicle (0.8% DMSO) to the 2× NCoR ID1- or ID2-bound spectra was performed using the minimal NMR chemical shift method (*42*). Peaks were identified to have broadened to zero if there was no confident peak in proximity to the vehicle peak. The average CSP and the SD in the CSPs were calculated for NCoR ID1 or ID2 titrations, and the peaks that displayed CSPs in the presence of peptide >1 SD above the average CSP were noted as significant. NMR CSP and peak intensity information is provided in data file S1.

### NCoR RID expression and purification

NCoR RID constructs (including WT, ΔID1, ΔID2, and ΔID1ΔID2) were transformed into BL21(DE3) *E. coli* cells, which were grown in 2 liters of Terrific Broth media [yeast extract (24 g/liter), tryptone (12 g/liter), KH_2_PO_4_ (23.1 g/liter), K_2_HPO_4_ (125.4 g/liter), and glycerol (4 ml/liter)] at 37°C until OD_600_ reached 0.7 to 0.8. Expression was induced with 0.75 mM IPTG for 2 hours at 37°C before cells were harvested by centrifugation. Pellets were washed 1× with phosphate-buffered saline and stored at −80°C until purification. Pellets were resuspended on ice in Hepes lysis buffer [25 mM Hepes (pH 7.4), 150 mM NaCl, 2 M urea, and 15 mM imidazole] supplemented with 1 mM DTT, pepstatin A, leupeptin, lysozyme, DNase I, phenylmethylsulfonyl fluoride, and 0.1% Tween 20, and cell slurry was sonicated on ice. Lysed cells were centrifuged at 20,000*g* for 30 min at 4°C, and soluble lysate was filtered before IMAC purification using 2 × 5 ml HisTrap columns (GE Healthcare) affixed to an ÄKTA pure; elution buffer was 25 mM Hepes (pH 7.4), 150 mM NaCl, and 500 mM imidazole. Fractions were pooled; additional protease inhibitors were added, and protein was dialyzed against 25 mM Hepes (pH 7.0), 150 mM NaCl, and 1 mM DTT with Tobacco etch virus (TEV) protease to cleave the Trx-6xHis tag at 4°C overnight. Cleaved protein was reloaded on HisTrap columns and eluted with 50 mM bis-tris (pH 6.7), 150 mM NaCl, and 1 mM DTT. Pooled flow-through fractions were concentrated at 4°C using centrifugal concentrators before size exclusion chromatography using a Superdex 75 column and a Hepes gel filtration buffer. Purity was confirmed to be ~85% by SDS-PAGE.

### Chemical cross-linking mass spectrometry

#### Sample preparation

Apo or heme-bound REV-ERBβ LBD and NCoR RID protein samples were cross-linked in Hepes gel filtration buffer [25 mM Hepes (pH 7.5) at room temperature, 50 mM NaCl, and 0.5 mM TCEP]. Non–cross-linked negative controls were generated using the same procedure without the addition of DSSO (Thermo Fisher Scientific). Samples included 4 μM apo LBD with or without 2 μM RID and 4 μM heme-bound LBD with or without 2 μM RID; complexes were preincubated 1 hour on ice. Reactions were initiated by spiking DSSO in DMSO at a final concentration of 600 μM in 50-μl solutions. The final molar ratio of cross-linker:protein was 100:1. The reactions were incubated at 25°C for 45 min before being quenched by the addition of tris (pH 8.0) to a final concentration of 50 mM. Each cross-link reaction was done in triplicate, and the reaction replicates were pooled after quenching. Samples (10 μl) were loaded for SDS-PAGE analysis with Coomassie staining to confirm the presence of cross-linked protein. The remaining cross-linked and non–cross-linked samples were then acetone-precipitated at −20°C overnight, and the protein was pelleted by centrifugation at 16,000 relative centrifugal force (rcf) for 5 min at 4°C. After decanting, the pellets were dried for 15 min at room temperature before being resuspended in 12.5 μl of resuspension buffer [50 mM ammonium bicarbonate and 8 M urea (pH 8.0)]. ProteaseMAX (Promega) was added to 0.02%, and the solutions were mixed on an orbital shaker operating at 400 rpm for 5 min. After resuspension, 87.5 μl of digestion buffer [50 mM ammonium bicarbonate (pH 8.0)] was added. Protein solutions were digested using trypsin (Promega) at a ratio of 1:200 (w/w, trypsin:total protein) and incubated at 37°C. After overnight incubation at 37°C, the samples were acidified by the addition of trifluoroacetic acid (TFA; Thermo Fisher Scientific) to 1%. Samples were then frozen and stored at −20°C until analysis.

#### Liquid chromatography and MS

Peptide samples were thawed, vortexed, and spun down at 16,000 rcf for 5 min. Ten microliters of peptide samples were loaded in an UltiMate 3000 autosampler (Dionex, Thermo Fisher Scientific). Approximately 500 ng of each peptide sample was injected in triplicate. Peptides were trapped on a μPAC trapping column (PharmaFluidics) using a load pump operating at 20 μl/min. Load pump buffer contained 2% acetonitrile and 0.1% TFA. After a 3-min desalting period, peptides were then separated using linear gradients (2 to 30% solvent B over 1 to 60 min and 30 to 95% solvent B over 60 to 90 min) at 1 μl/min on a 50-cm μPAC C18 column (PharmaFluidics). Gradient solvent A contained 0.1% formic acid, and solvent B contained 80% acetonitrile and 0.1% formic acid. The liquid chromatography eluate was interfaced to an Orbitrap Fusion Lumos (Thermo Fisher Scientific) via nanospray ionization source. Cross-links were identified using a previously described method (*43*). Master scans of mass/charge ratio (*m*/*z*) 375 to 1500 were taken in the Orbitrap mass analyzer operating at 60,000 resolution at 400 *m*/*z*, with automatic gain control target set to 400,000. Maximum ion injection time was set to 50 ms, and advanced peak detection was enabled. Precursor ions with a charge state between 4 and 8 were selected via quadrupole for collision-induced dissociation fragmentation at 25% collision energy and 10-ms reaction time. Fragment ion mass spectra were taken on the Orbitrap mass analyzer operating at 30,000 resolution at 400 *m*/*z*, with an automatic gain control target of 50,000, and maximum injection time was set to 150 ms. Doublet pairs of ions with the targeted mass difference for sulfoxide fragmentation [31.9721 Da (*44*)] were selected for higher-energy collisional dissociation fragmentation at 35% collision energy. MS3 scans were collected in the ion trap operating in “rapid” mode at automatic gain control target and maximum ion injection time set to 20,000 and 200 ms, respectively.

#### Data analysis

Cross-links were identified using the XlinkX algorithm (*45*) implemented on Proteome Discoverer (version 2.2). Cross-links were considered for lysine, threonine, serine, and tyrosine residues, and the validation strategy was set to “simple” where relaxed and strict false discovery rates were set to 0.05 and 0.01, respectively. During database searches, the target databases only contained sequences for the proteins that were analyzed, and the decoy database contained the reverse sequences. The maximum missed cleavages were set to 8. Methionine oxidation was considered as a variable modification. Mass tolerances for precursor Fourier transform MS (FTMS), fragment FTMS, and fragment ion trap MS (ITMS) were set to 10 and 20 parts per million (ppm) and 0.5 Da, respectively. Peak areas for identified cross-links were quantified using Skyline (version 19.1) using a previously described protocol (*46*). Cross-link spectral matches found in Proteome Discoverer were exported and converted to sequence spectrum list format using Excel (Microsoft). Cross-link peak areas were assessed using the MS1 full-scan filtering protocol for peaks within 8 min of the cross-link spectral match identification. Peaks areas were assigned to the specified cross-linked peptide identification if the mass error was within 10 ppm of the theoretical mass, if the isotope dot product was greater than 0.95, and if the peak was not found in the non–cross-linked negative controls. Pairwise comparisons were made using “MSstats” package (*47*) implemented in the Skyline browser to calculate relative fold changes and significance (multiple testing adjusted *P* values). Significant changes were defined as −log_10_; adjusted *P* value was greater than 1.3 (*P* value less than 0.05). The results from skyline were exported and are reported in an Excel spreadsheet. Dot plots were generated in R. SASD calculations were determined using Jwalk (*48*) using the heme-bound LBD structure as a template (PDB 3CQV). Analyzed XL-MS data are provided in data file S2.

### Bmal1-luciferase assay

HEK293T cells were plated at 1.5 × 10^3^ cells per well in 96-well flat-bottom plates in DMEM 24 hours before transfection. Transfections were performed using TransIT-LT1 transfection reagent (Mirus Bio) using 3 μl of reagent/1 μg of DNA. pGL3-Bmal1-luciferase construct (100 ng; WT, pRORE mutant, dRORE mutant, or p/dRORE mutant) and pcDNA3.1 construct (25 ng; empty vector or REV-ERBβ) were cotransfected per well (six replicate wells per condition). Luciferase activity was harvested 24 hours after transfection with Britelite Plus (PerkinElmer) and read using a BioTek Synergy plate reader. Raw luminescence values of REV-ERBβ–transfected wells were normalized to pcDNA3.1 empty vector transfection wells individually for each *Bmal1*-luciferase construct to determine fold repression by REV-ERBβ for each *Bmal1-*luciferase construct. Student’s *t* test was used for comparison between groups, and a *P* < 0.05 was considered statistically significant. Normalization and statistical analysis were performed using GraphPad Prism 8. *n* = 3 independent experiments were performed.

## SUPPLEMENTARY MATERIALS

Supplementary material for this article is available at http://advances.sciencemag.org/cgi/content/full/7/5/eabc6479/DC1

## Appendix

View/request a protocol for this paper from *Bio-protocol*.
